# Development and Validation of Human-AI Collaborative Workflow in SNOMED CT Mapping of Bilingual Clinical Text

**DOI:** 10.1007/s10916-026-02465-3

**Published:** 2026-10-02

**Authors:** Hyeonhoon Lee, Seonhye Choi, Duyeon Kim, Kyunglan Hong, Hyeonsik Kim, Chang Wook Jeong, Hyung-Chul Lee

**Affiliations:** 1 Department of Transdisciplinary Medicine, Seoul National University Hospital, Seoul, Republic of Korea; 2 Healthcare AI Research Institute, Seoul National University Hospital, Seoul, South Korea; 3 Department of Medicine, Seoul National University College of Medicine, Seoul, South Korea; 4 Office of Hospital Information, Seoul National University Hospital, Seoul, South Korea; 5 Department of Interdisciplinary Program in Medical Informatics, Seoul National University, Seoul, Republic of Korea; 6 Department of Anesthesiology and Pain Medicine, Seoul National University College of Medicine, Seoul National University Hospital, 101 Daehak-ro, Jongno-gu, Seoul, 03080 Republic of Korea

**Keywords:** SNOMED CT, Clinical terminology mapping, Large language models, Retrieval-augmented generation, Health information exchange

## Abstract

Systematized Nomenclature of Medicine–Clinical Terminology (SNOMED CT) is the principal international standard for semantic interoperability of clinical information, but mapping free-text clinical narratives to SNOMED CT concepts remains labor-intensive. We developed a large language model agent system for mapping bilingual clinical text to SNOMED CT concepts and evaluated its effect on mapping accuracy and efficiency within a human-AI collaborative workflow. We designed a three-module agent system comprising translation, abbreviation expansion, and vector-based retrieval components, integrated with a pre-embedded SNOMED CT vector database. Three health information managers independently mapped bilingual clinical text segments using three approaches: human-only, Agent-only, and Agent-assisted human mapping. Performance was evaluated by using hit rate, precision, recall, and F1 score at k = 1 and 5, and R-precision. Mapping time was compared between human-only and human-AI collaborative approaches. A total of 2,261 de-identified clinical text segments across nine clinical categories were collected at a tertiary academic hospital in South Korea. The human-AI collaborative workflow, which expanded the set of valid SNOMED CT candidates presented at each mapping decision, raised pooled hit rate@1 from 0.837 to 0.868 (difference 0.031, 95% confidence interval [CI] 0.021 to 0.042; *p* < 0.001), raised R-precision from 0.632 to 0.674 (difference 0.042, 95% CI 0.034 to 0.051; *p* < 0.001), and reduced total mapping time by 53.9% (from 1.57 to 0.72 min per segment, including agent processing). By expanding the space of valid SNOMED CT candidates available to expert mappers, the human-AI collaborative approach improved SNOMED CT mapping accuracy while reducing time by about half. Its modular architecture, supporting periodic vector database updates without retraining, offers a sustainable and efficient solution for bilingual clinical terminology standardization.

**Supplementary Information:**

The online version contains supplementary material available at https://doi.org/10.1007/s10916-026-02465-3.

## Introduction

The Systematized Nomenclature of Medicine–Clinical Terminology (SNOMED CT) is a comprehensive, multilingual clinical terminology comprising 368,285 active concepts for the consistent and computable representation of healthcare information [1], with a semantic network of hierarchical and associative relationships across diagnoses, procedures, and findings [2]. It is widely adopted internationally as a foundation for semantic interoperability across electronic health records, clinical decision support systems, and public health infrastructures [3, 4]. Standardized terminology underpins consistent documentation, multi-center data integration, and secondary use of health data for research. With the increasing use of artificial intelligence (AI) in clinical settings, the demand for large-scale, semantically normalized, machine-interpretable data has grown rapidly [5, 6], and SNOMED CT addresses this need by consolidating diverse clinical expressions into unified concepts [7].

Despite these structural advantages, mapping clinical text to SNOMED CT remains challenging. Manual annotation is labor-intensive and frequently suffers from inter-annotator variability, especially when clinical narratives are ambiguous or context-dependent [8]; a comparative study of three professional coding services found highly inconsistent semantic agreement in concept assignment [9], and such inconsistencies can propagate errors into downstream systems and hinder reproducibility [3, 10]. These challenges motivate scalable and reproducible concept mapping frameworks [11].

Various approaches have been explored for automated terminology standardization. Deep-learning approahces outperform word-level matching for terminology mapping in the Observational Medical Outcomes Partnership (OMOP) common data model [12]; a bidirectional encoder representations from transformers (BERT)-based two-stage entity linking method has been proposed for SNOMED CT [13]; large language model (LLM)-generated embeddings achieved a 96% top-three alignment rate for flowsheet concept mapping [14]; standalone LLMs perform poorly as medical coders without retrieval-based augmentation [15]; and transformer models have been used to detect missing is-a relationships within SNOMED CT [16]. Most of these studies pursue fully automated workflows intended to replace manual coding entirely; however, the semantic and hierarchical structure of SNOMED CT still requires expert judgment informed by concept relationships and clinical context, making the SNOMED CT Browser-based workflow essential for reliable mapping in practice.

In this study, we aimed to develop and evaluate an agent system for supporting SNOMED CT mapping of bilingual clinical text, with attention to both mapping accuracy and coding efficiency. Using 2,261 clinical text segments from a tertiary academic hospital, we compared three mapping strategies: human-only, Agent-only, and Agent-assisted human mapping. We hypothesized that the Agent-assisted approach would improve mapping accuracy and coding efficiency compared with manual mapping.

## Methods

### Data Sources and Collection

Clinical text data were derived from the Seoul National University Hospital (SNUH) complex disease assessment form, a structured form used for inpatient severity classification. Under the Korean diagnosis-related group system, discharged inpatients classified into general or simple disease groups undergo additional evaluation by attending clinicians to determine whether the case qualifies as a complex disease requiring tertiary-level care [17]. Clinicians document free-text justifications for this classification across nine predefined categories: test, advanced procedure, advanced treatment, underlying disease, lesion characteristics, medication, recurrent disease, symptom, and medical history. Each such free-text entry constitutes a single clinical text segment, which is the unit of analysis in this study. Free-text entries recorded between March 2022 and June 2024 were retrieved from the SNUH Integrated Indicator Management System (SIMS) across all nine categories. Only discharges for which a clinician had recorded at least one free-text justification were retained, and entries were sampled for balanced coverage of the categories. Duplicate entries or those with excessively short or long text were excluded. These exclusions were applied when the dataset was assembled; no segment was excluded on the basis of its mapping outcome. Each clinical text segment was annotated with a category and a department label.

### SNOMED CT Reference Database

For similarity-based retrieval, we utilized the SNOMED CT International Edition (version 2024-08-01), released 1 August 2024 [18]. SNOMED CT concepts and their associated descriptions were pre-embedded in a vector database (ChromaDB) for semantic similarity operations [19]. The database encompassed concept identifiers, fully specified names, and textual labels across clinical finding, procedure, and disorder domains.

### Agent System Architecture

**Fig. 1:**
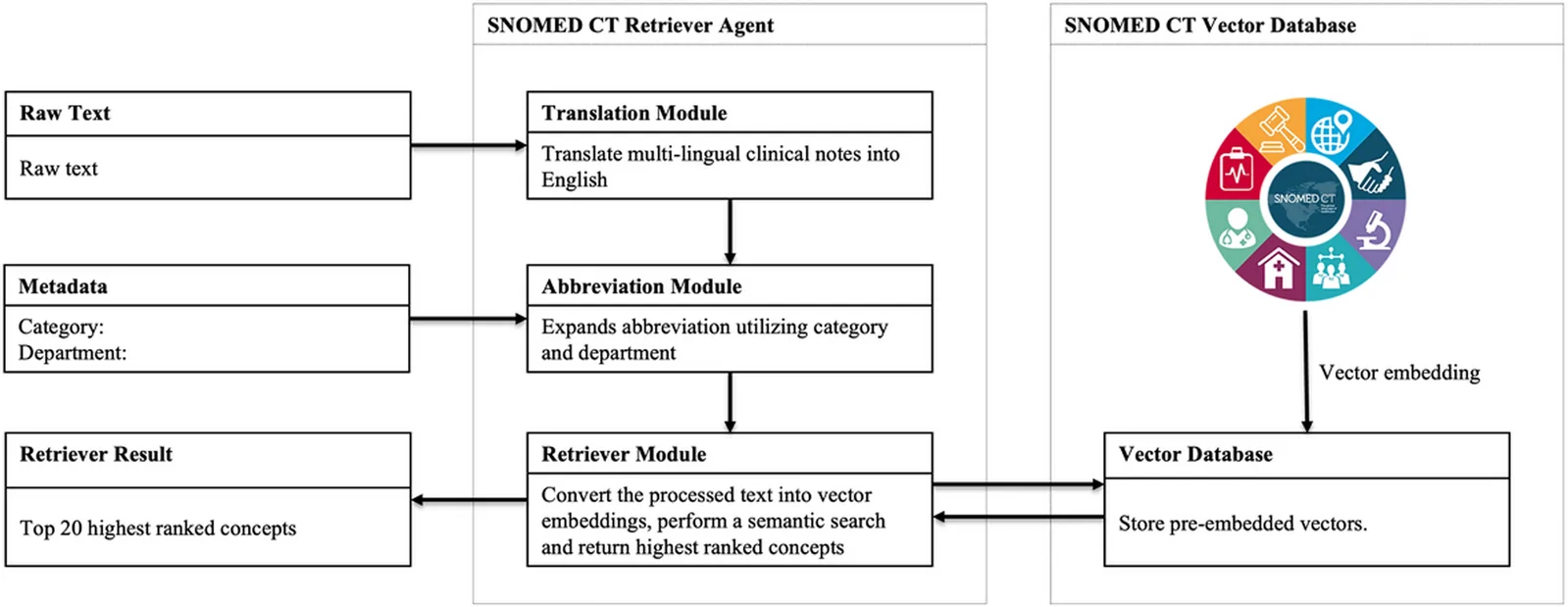
Architecture of the vector-based retrieval large language model (LLM) agent system for SNOMED CT mapping. Free-text clinical input and accompanying metadata (category, department) are first processed by the Translation Module, which converts non-English text to English. The Abbreviation Expansion Module then resolves domain-specific acronyms using the supplied metadata. The normalized text is passed to the Retriever Module, which embeds the text, performs a cosine similarity search against a vector database (ChromaDB) containing pre-embedded SNOMED CT concept representations, and returns the top-20 ranked candidate concepts. The candidate list is then presented to health information managers for final selection in the LLM-assisted workflow. SNOMED CT concept embeddings are generated offline and refreshed periodically to align with terminology updates

We designed an agent-based system built on the LangChain framework using GPT-4 Turbo as the underlying LLM. The system accepts raw clinical text and sequentially processes it through three modules: (1) a Translation Module, (2) an Abbreviation Expansion Module, and (3) a Retriever Module, with the category and department labels annotated during data collection serving as contextual metadata (Fig. 1).

#### Translation Module

The Translation Module detects the language of the input text and translates non-English passages, including Korean-only or mixed Korean–English, into English. Clinical terms, drug names, and procedure names already written in English were preserved to maintain medical specificity. The module was implemented as a LangChain ReAct agent (langchain v0.1.20) driven by OpenAI GPT-4 Turbo (gpt-4-turbo, temperature = 0), with Google Search and Bing Search exposed as tools through SerpAPIWrapper (google-search-results v2.4.2) for consulting external evidence on ambiguous phrases. The complete system prompt, including the few-shot examples that constrain output to translation only, is provided in Supplementary Material 1.

#### Abbreviation Expansion Module

The Abbreviation Expansion Module identifies and expands domain-specific abbreviations and acronyms by leveraging category and department metadata. For example, "PCI" is expanded to "Percutaneous Coronary Intervention" when the department is Cardiology, but may be expanded differently in other clinical contexts. The module identifies potential abbreviations by treating capitalization as a probabilistic cue rather than a filter, so lowercase and mixed-case abbreviations are also expanded, and uses LLM-based prompts augmented with web search tools to retrieve context-appropriate full forms when the category and department metadata alone do not determine the expansion. The complete system prompt, which directs the module to expand abbreviations into the form "Expanded Phrase (ABBR)" rather than narrative interpretations, is provided in Supplementary Material 1.

#### Retriever Module

The Retriever Module encodes the processed text using the MedCPT-Query-Encoder [20], a biomedical domain-specific transformer model, and performs a cosine similarity search against the pre-embedded SNOMED CT concept database. The module returns the top 20 candidate SNOMED CT concepts (concept identifier and fully specified name) for subsequent evaluation or human review.

### Experimental Design

Three mapping approaches were evaluated on the same set of de-identified clinical text segments. Mappers A–C performed the human-only condition over one month in September 2024 and the Agent-assisted condition over one month in February 2025, in a fixed order, separated by a wash-out interval of approximately four months. In both conditions, segments were presented as independent items without access to other segments from the same discharge case, with only their category and department labels.

#### Agent-only

The full agent pipeline (Translation Module, Abbreviation Expansion Module, and Retriever Module) was executed without human intervention. The top 20 candidate SNOMED CT concepts returned by the Retriever Module were recorded as the final output.

#### Human-only

Three health information managers (Mappers A, B, and C) independently performed manual coding using the SNOMED CT Browser without access to any agent-generated suggestions. Browser-based search procedures are described in Supplementary Material 2.

#### Agent-assisted human

**Fig. 2:**
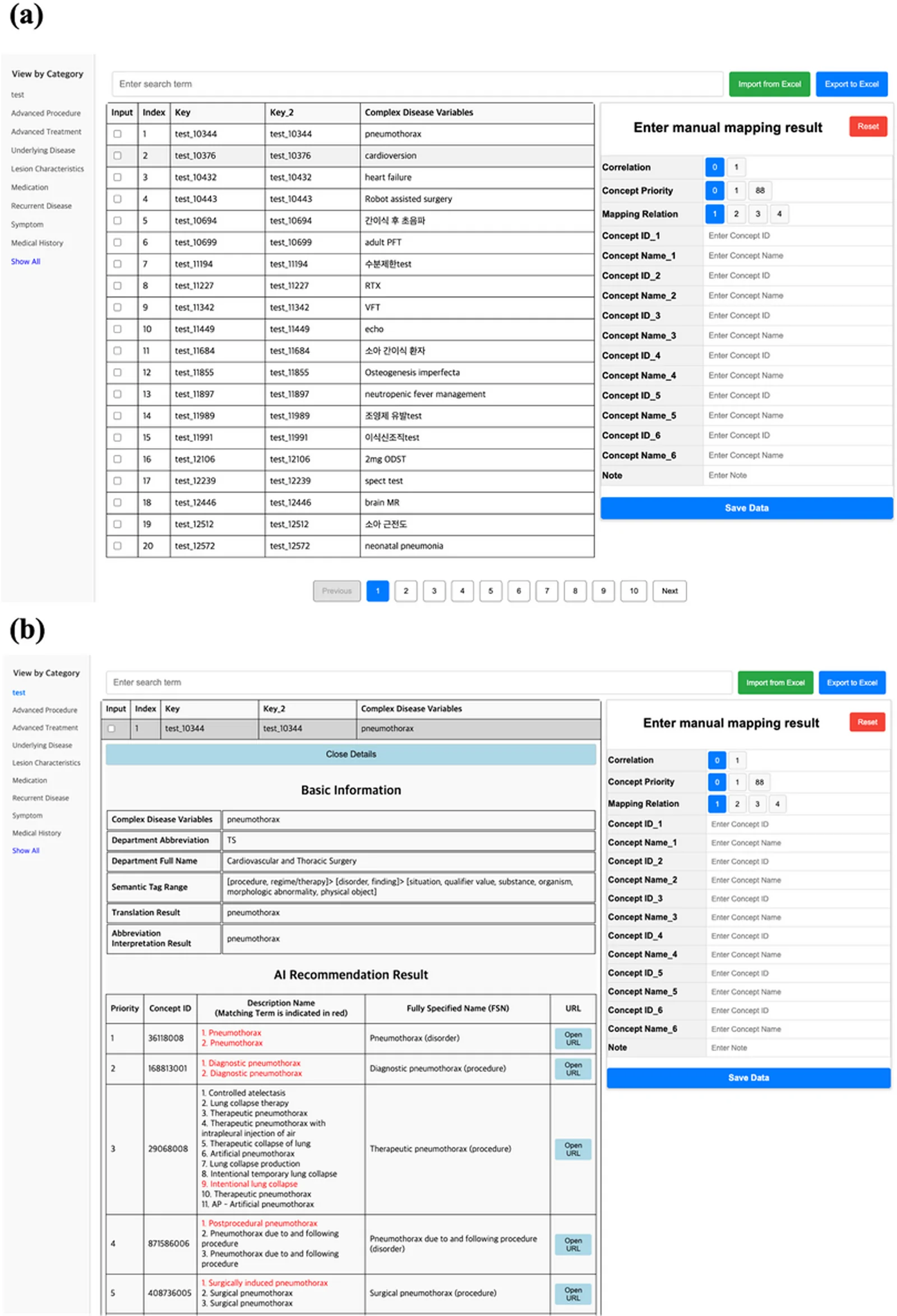
Web-based mapping interface for large language model-assisted human coding.(**a**) The mapping dashboard lists clinical text segments with filterable categories (left panel) and provides a manual entry panel (right panel) where health information managers can enter or batch-import/export up to six SNOMED CT Concept IDs and fully specified names. (**b**) The detail view for a selected text segment displays the normalized text, metadata, and a ranked list of agent-suggested SNOMED CT candidates. Exact term matches are highlighted, and each candidate row supports one-click copying of the Concept ID and opening of the concept in the external SNOMED CT Browser

A web-based interface presented the agent's SNOMED CT candidates to the three health information managers, who retained full authority to select, modify, or reject them; it included category-based filtering, manual entry, exact-match highlighting, and direct integration with the external SNOMED CT Browser (Figure 2). Where a segment expressed more than one concept, mappers were instructed to select every applicable SNOMED CT concept rather than a single best concept, using the six concept slots provided by the interface (Fig. 2a).

### Reference Set Establishment

The reference set was established by an independent reference panel of three professional health information managers who were not involved in any of the subsequent mapping experiments. Each panel member independently reviewed every segment and assigned SNOMED CT concepts using the SNOMED CT Browser. The three independent assignments were consolidated into a consensus reference label through panel discussion, which constituted the first adjudication round. In a second round, a physician who is a professor of medical informatics joined the panel, and all segments were adjudicated again under the same procedure. Both rounds were conducted blind to the experimental origin of the candidate concepts, and only the consensus reached in the second round was retained as the reference set. Mappers A, B, and C, who performed the Human-only and Agent-assisted conditions (see Experimental design), formed a separate group, had no role in establishing the reference set, and remained blind to the consensus labels throughout. Where no exactly equivalent concept existed, the panel assigned the nearest broader concept, recorded as a narrow-to-broad map correlation; consequently, all segments received at least one reference concept, and none was classified as not mappable [3].

### Evaluation Metrics

Mapping performance was evaluated using four retrieval metrics computed at k = 1 and k= 5, where k denotes the number of top-ranked candidates considered, and R-precision, which evaluates each segment at a cutoff equal to the number of its reference concepts [21–23]. For each clinical text segment, the set of reference SNOMED CT concepts was compared with the top-k codes returned by the system or selected by the mapper.

Hit rate@k measures whether at least one correct code appears among the top-k candidates, recorded as a binary success for each segment. Precision@k is defined as the proportion of the k retrieved codes that are relevant to the reference set. Recall@k is the fraction of all reference concepts that are successfully retrieved within the top-k candidates. F1@k is the harmonic mean of precision@k and recall@k, providing a balanced measure of retrieval quality. R-precision is the fraction of a segment's g reference concepts that appear among the first g codes returned or selected; because the cutoff adapts to g, a perfect mapping scores 1.0 on every segment, and for single-concept segments it coincides with hit rate@1. For the human conditions, concepts were taken in the order recorded. All metrics were reported as macro-averages across all evaluated segments. For a segment with g reference concepts, recall@1 is bounded above by 1/g and cannot reach 1.0 for multi-concept segments; conversely, precision@k divides by k irrespective of g. Hit rate@k and precision@1 are not subject to these bounds.

All metrics were reported independently for each mapper under each condition. The pairwise Jaccard index was computed between each pair of mappers (A–B, A–C, B–C) at the segment level as |X ∩ Y|/|X ∪ Y|, where X and Y denote the sets of SNOMED CT concepts selected by the two mappers for the same clinical text segment. The three pairwise values were averaged per segment and across segments, giving a measure of concept-selection overlap independent of accuracy. When both mappers selected no concepts the index was defined as 1.0, and when only one did, as 0.0.

### Mapping Time Measurement

SNOMED CT mapping time was recorded for each mapper across all nine clinical categories in both conditions. The web-based interface automatically logged cumulative time spent reviewing and selecting codes in the Agent-assisted condition, whereas mappers self-reported total coding time in the human-only condition. Mean mapping time per segment was calculated for each mapper and condition.

### Subgroup Analyses

Subgroup analyses examined performance by number of SNOMED CT concepts per record (single versus multiple) and by clinical category.

### Statistical Analysis

All analyses were performed using Python (version 3.9.6) with the packages NumPy (version 1.26.4), pandas (version 2.2.2), and SciPy (version 1.13.1). Means with 95% confidence intervals (CIs) for all performance metrics were computed using non-parametric bootstrap resampling with 10,000 iterations. To compare performance across mapping approaches, paired t-tests were conducted for three pairwise comparisons. Mapping-time reductions were assessed with the Wilcoxon signed-rank test, paired by mapper and clinical category, overall and per mapper. Statistical significance was set at p < 0.05, with Bonferroni correction for multiple comparisons.

## Results

### Dataset Characteristics

**Fig. 3:**
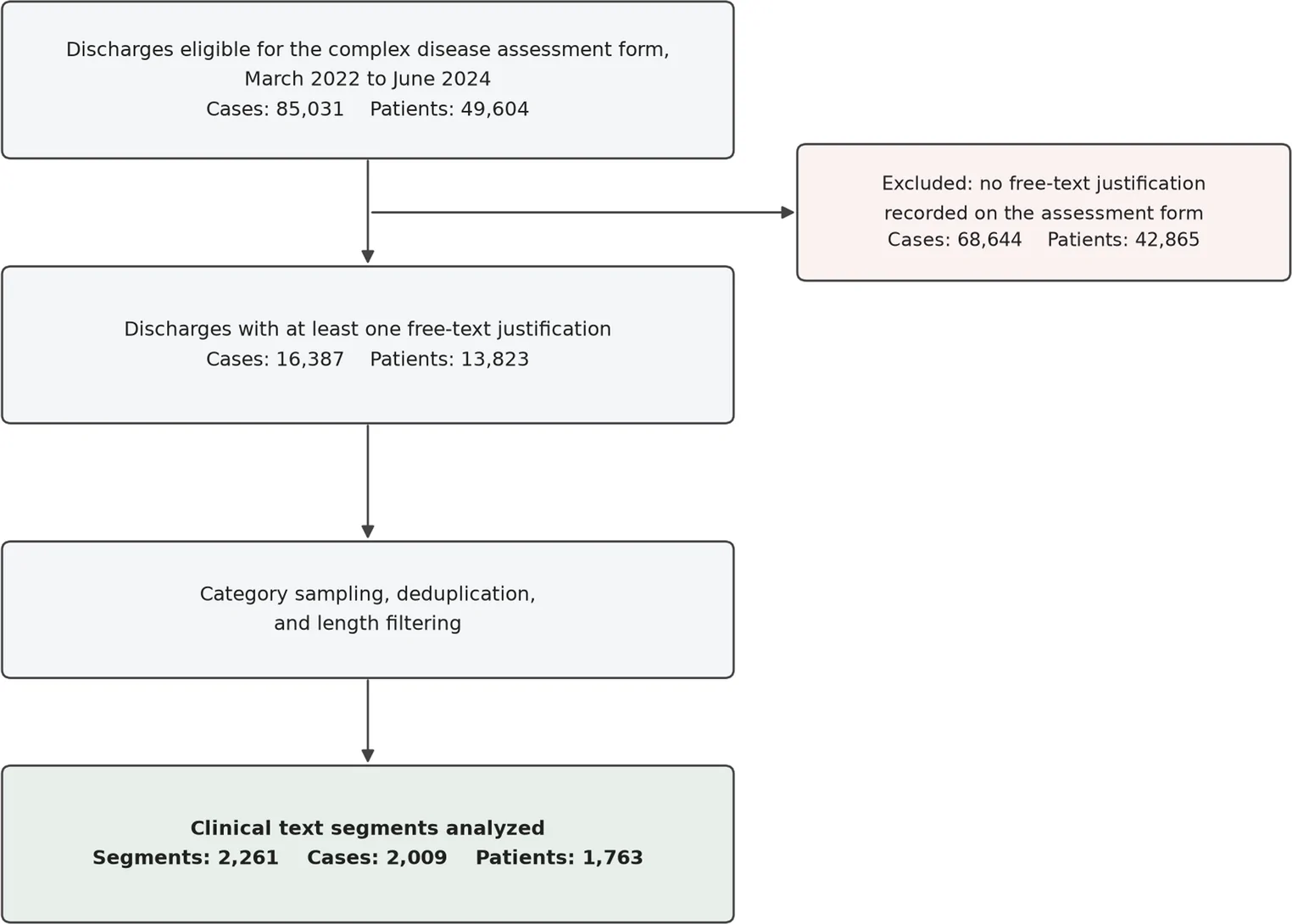
Cohort selection.Of 85,031 discharge cases (49,604 patients) eligible for the complex disease assessment form at Seoul National University Hospital between March 2022 and June 2024, 68,644 had no free-text justification recorded, leaving 16,387 cases (13,823 patients). Free-text entries were collected across the nine categories and sampled for balanced category coverage, and duplicates and excessively short or long entries were excluded, yielding 2,261 clinical text segments from 2,009 discharge cases and 1,763 distinct patients

**Fig. 4:**
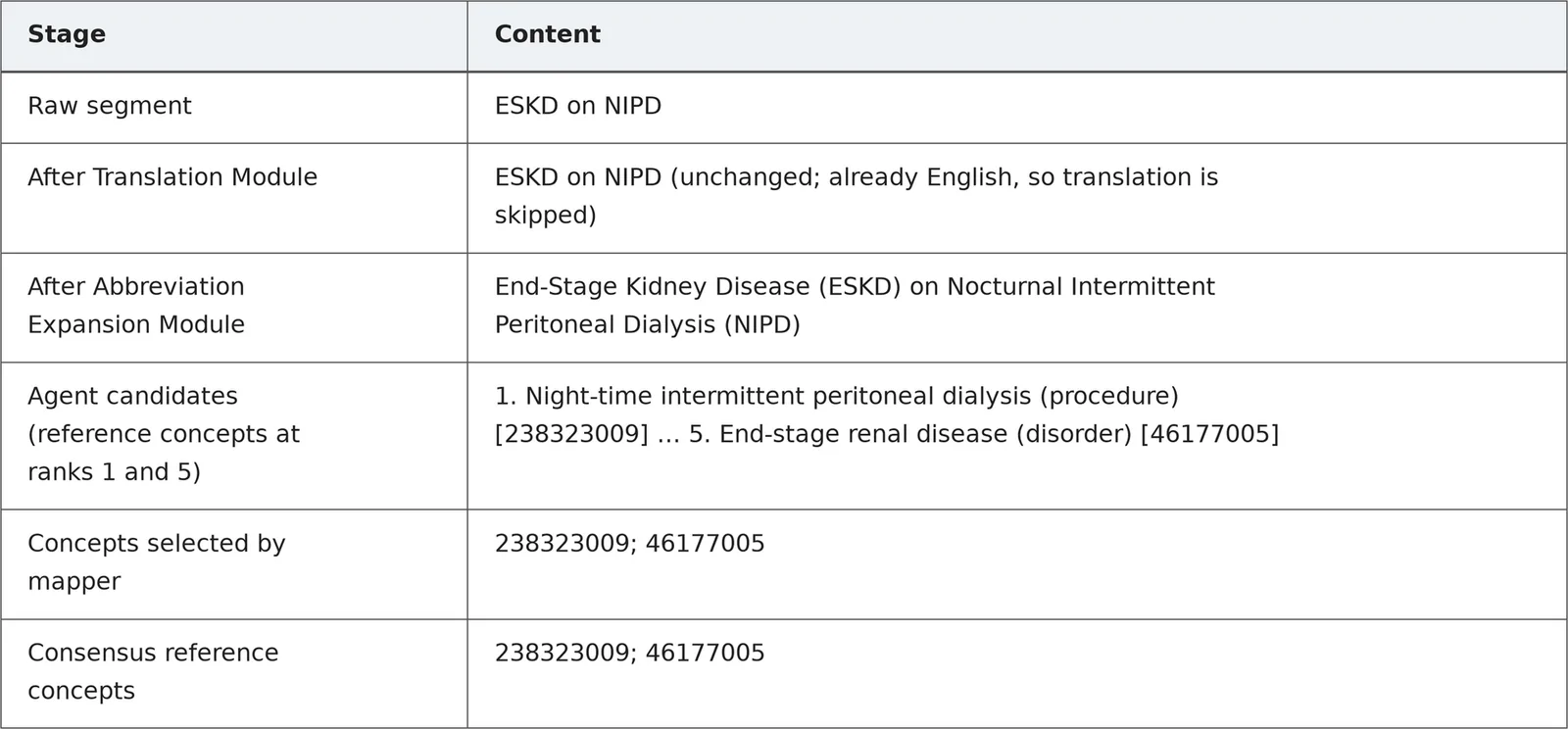
Worked example of one clinical text segment processed end-to-end. The segment "ESKD on NIPD", drawn from the test category, carries two reference concepts. The Translation Module leaves the segment unchanged because it is already in English, the Abbreviation Expansion Module resolves both abbreviations, and the Retriever Module returns both reference concepts among the top-ranked candidates, at ranks 1 and 5

Of 85,031 discharges eligible for the complex disease assessment form between March 2022 and June 2024, 16,387 (13,823 patients) contained at least one free-text justification (Fig. 3). After excluding duplicate entries and those with excessively short or long text, the final dataset comprised 2,261 de-identified clinical text segments originating from 2,009 discharge cases and 1,763 distinct patients. Table 1 summarizes dataset characteristics. The segments spanned nine clinical categories (250–254 segments per category), and covered 58 clinical departments, most frequently orthopedic surgery (14.4%). According to the reference set, 1,107 segments (49.0%) were mapped to a single SNOMED CT concept, and 1,154 segments (51.0%) were mapped to multiple concepts, with a median of 2 concepts per segment. Based on the semantic tag appended to each fully specified name, the mapped SNOMED CT concepts were predominantly procedures (37.9%) and disorders (35.0%), followed by clinical findings (8.5%) and substances (6.8%). A representative segment processed end-to-end is shown in Fig. 4. The segments were short, with a median of 12 characters (interquartile range [IQR] 7–18) and two whitespace-delimited words (IQR 1–3) per segment; length by clinical category is given in Supplementary Material 3.

**Table 1:** Dataset characteristics

Characteristic	Value (*n*, %)
*Clinical categories (segments)*	
Test	250 (11.1%)
Advanced procedure	250 (11.1%)
Advanced treatment	254 (11.2%)
Underlying disease	254 (11.2%)
Lesion characteristics	252 (11.1%)
Medication	250 (11.1%)
Recurrent disease	250 (11.1%)
Symptom	250 (11.1%)
Medical history	251 (11.1%)
*Number of concepts per segment*	
Single concept	1,107 (49.0%)
Multiple concepts	1,154 (51.0%)
Median (range)	2 (1–11)
Total SNOMED CT concepts	4,264
*Segment length*	
Characters, median (IQR)	12 (7–18)
Characters, range	2–67
Words, median (IQR)	2 (1–3)
Words, range	1–13
*Semantic tag distribution (concepts)*	
Procedure	1,616 (37.9%)
Disorder	1,494 (35.0%)
Clinical finding	362 (8.5%)
Substance	290 (6.8%)
Other	502 (11.8%)
*Clinical departments (segments)*	
Orthopedic surgery	325 (14.4%)
Pediatric orthopedic surgery	239 (10.6%)
Cardiovascular and thoracic surgery	164 (7.3%)
Hepatobiliary and pancreatic surgery	76 (3.4%)
Nephrology	75 (3.3%)
Ophthalmology	75 (3.3%)
Pediatrics (neurology)	70 (3.1%)
Hematology and oncology	70 (3.1%)
Pediatrics (nephrology)	67 (3.0%)
Obstetrics and gynecology	58 (2.6%)
Others (48 departments)	1,042 (46.1%)

### Overall Performance Comparison

Table 2 presents the performance of the three mapping approaches at k = 1 and k = 5, together with R-precision. The Agent-assisted human approach achieved a pooled hit rate@1 of 0.868 (95% CI, 0.860–0.876), outperforming both the Agent-only approach (0.701; 95% CI, 0.682–0.720) and the pooled human-only performance (0.837; 95% CI, 0.828–0.845). At k = 5, Agent-assisted mapping maintained a pooled hit rate@5 of 0.900 (95% CI, 0.893–0.907), compared with 0.874 (95% CI, 0.860–0.887) for Agent-only and 0.869 (95% CI, 0.861–0.877) for pooled human-only. Under R-precision, Agent-assisted mapping scored 0.674 (95% CI, 0.666–0.682), against 0.632 (95% CI, 0.623–0.640) for pooled human-only and 0.620 (95% CI, 0.603–0.636) for Agent-only.

**Table 2:** Performance comparison across three mapping approaches at fixed cutoff metrics k = 1 and k = 5, and R-precision

Experiment	Hit Rate@1	Precision@1	Recall@1	F1@1	Hit Rate@5	Precision@5	Recall@5	F1@5	*R*-precision
Human-only (Mapper A)	0.826 (0.810–0.841)	0.826 (0.810–0.841)	0.577 (0.562–0.593)	0.645 (0.630–0.660)	0.877 (0.863–0.890)	0.209 (0.204–0.214)	0.659 (0.645–0.674)	0.301 (0.295–0.307)	0.636 (0.621–0.651)
Human-only (Mapper B)	0.852 (0.837–0.866)	0.852 (0.837–0.866)	0.589 (0.574–0.604)	0.661 (0.647–0.675)	0.874 (0.860–0.887)	0.200 (0.196–0.204)	0.642 (0.627–0.657)	0.289 (0.283–0.295)	0.637 (0.622–0.652)
Human-only (Mapper C)	0.832 (0.816–0.847)	0.832 (0.816–0.847)	0.579 (0.563–0.594)	0.648 (0.634–0.663)	0.856 (0.841–0.870)	0.194 (0.189–0.198)	0.626 (0.611–0.641)	0.281 (0.275–0.286)	0.623 (0.608–0.638)
Human-only (Pooled)	0.837 (0.828–0.845)	0.837 (0.828–0.845)	0.582 (0.573–0.591)	0.652 (0.643–0.660)	0.869 (0.861–0.877)	0.201 (0.198–0.204)	0.642 (0.634–0.651)	0.290 (0.287–0.293)	0.632 (0.623–0.640)
Agent-only	0.701 (0.682–0.720)	0.701 (0.682–0.720)	0.505 (0.488–0.522)	0.559 (0.542–0.576)	0.874 (0.860–0.887)	0.243 (0.237–0.249)	0.734 (0.719–0.749)	0.345 (0.338–0.353)	0.620 (0.603–0.636)
Agent-assisted (Mapper A)	0.839 (0.824–0.854)	0.839 (0.824–0.854)	0.586 (0.570–0.601)	0.655 (0.640–0.669)	0.860 (0.845–0.874)	0.190 (0.186–0.195)	0.627 (0.611–0.642)	0.278 (0.272–0.283)	0.625 (0.610–0.641)
Agent-assisted (Mapper B)	0.907 (0.895–0.919)	0.907 (0.895–0.919)	0.631 (0.617–0.646)	0.707 (0.694–0.720)	0.916 (0.904–0.927)	0.203 (0.200–0.207)	0.671 (0.657–0.685)	0.297 (0.292–0.302)	0.670 (0.655–0.684)
Agent-assisted (Mapper C)	0.858 (0.843–0.872)	0.858 (0.843–0.872)	0.600 (0.584–0.615)	0.670 (0.656–0.684)	0.924 (0.913–0.935)	0.254 (0.248–0.261)	0.760 (0.747–0.774)	0.360 (0.353–0.366)	0.727 (0.713–0.742)
Agent-assisted (Pooled)	0.868 (0.860–0.876)	0.868 (0.860–0.876)	0.606 (0.597–0.614)	0.677 (0.669–0.685)	0.900 (0.893–0.907)	0.216 (0.213–0.219)	0.686 (0.678–0.695)	0.311 (0.308–0.315)	0.674 (0.666–0.682)

Furthermore, agent-assisted mapping significantly outperformed both human-only and Agent-only approaches at k = 1 for all metrics (all *p* < 0.001). Pairwise comparisons among the three groups appear in Supplementary Material 4. The hit rate@1 difference was 0.031 (95% CI, 0.021–0.042) for Agent-assisted versus human-only and 0.167 (95% CI, 0.156–0.177) for Agent-assisted versus Agent-only. Agent-only performed significantly below pooled human-only in hit rate@1 (difference = − 0.135; 95% CI, − 0.148 to − 0.123; *p* < 0.001). At k = 5, Agent-only did not differ significantly from pooled human-only in hit rate, but exceeded it in precision@5, recall@5 and F1@5 (all *p* < 0.001), with a recall@5 difference of 0.092 (95% CI, 0.082–0.102). Under R-precision, the Agent-assisted advantage was 0.042 (95% CI, 0.034–0.051) over human-only and 0.054 (95% CI, 0.045–0.063) over Agent-only (both *p* < 0.001), while Agent-only did not differ significantly from pooled human-only (difference = − 0.012; corrected *p* = 0.070).

### Human–Agent Interaction Analysis

Concept-selection overlap among the three mappers, measured by the mean pairwise Jaccard index, was 0.915 (95% CI, 0.906–0.923) in the human-only condition and 0.639 (95% CI, 0.624–0.653) under assistance. Stratified by the number of reference concepts per segment, the Agent-assisted Jaccard was 0.836 (95% CI, 0.819–0.853) for single-concept and 0.450 (95% CI, 0.433–0.467) for multi-concept segments; the corresponding human-only values were 0.969 (95% CI, 0.961–0.977) and 0.862 (95% CI, 0.847–0.877).

To characterize how mappers used the agent’s candidate list, we analyzed the rank of correctly mapped concepts within the top-20 retrieval results. Among the 4,264 reference concepts, 3,224 (75.6%) appeared in the agent’s top-20 candidates; the highest-ranked correct concept appeared at rank 1 in 70.1% of segments, within the top 5 in 87.4%, and within the top 20 in 91.9%. About 79% of concepts selected by Agent-assisted mappers came from the candidate list, with a mean within-list rank of 3.1–3.7, and the remaining 21% were identified independently through the SNOMED CT Browser. Mapper C submitted the most concepts (3,723 versus 2,634 and 2,540), selected the agent’s top-1 candidate least often (34.8% versus 43.4% and 42.8%), and had the highest recall@5 (0.760 versus 0.627 and 0.671).

The mean total mapping time for 2,261 segments was 1,638.9 min (27.3 h) in the Agent-assisted condition, including 431.1 min of agent processing, versus 3,551.0 min (59.2 h) in the human-only condition, a 53.9% reduction (*p* < 0.001) (Table 3); per-mapper times were 1,737.1, 1,803.1, and 1,376.4 min (reductions of 51.7%, 53.1%, and 57.0%; all *p* = 0.004). The mean time per segment decreased from 1.57 to 0.53 min of clinician time; including the agent’s preprocessing, measured at 11.44 s per segment (95% CI 10.23–12.74; per-stage latency in Supplementary Material 5), the total was 0.72 min. Across categories, the greatest reduction was for symptoms (64.6%) and the smallest for advanced treatment (35.8%).

**Table 3:** SNOMED CT mapping time comparison between Agent-assisted and human-only approaches across clinical categories

Clinical Category	*N*	Agent-assisted (min/segment)	Human-only (min/segment)	Time reduction (%)
Test	250	0.96	1.75	45.4
Advanced procedure	250	0.91	2.13	57.3
Advanced treatment	254	0.88	1.36	35.8
Underlying disease	254	0.76	1.74	56.5
Lesion characteristics	252	0.69	1.54	55.0
Medication	250	0.64	1.32	51.8
Recurrent disease	250	0.57	1.45	60.4
Symptom	250	0.55	1.55	64.6
Medical history	251	0.57	1.29	55.7
Overall	2,261	0.72	1.57	53.9

### Subgroup Analysis

Table 4 presents the subgroup analysis for all three conditions at k = 5, together with R-precision. Performance differed between single- and multiple-concept records. Agent-assisted mapping exceeded human-only mapping on F1 in eight of the nine clinical categories and in both concepts-per-record strata. On R-precision, Agent-assisted mapping exceeded human-only mapping in both strata and in eight of the nine clinical categories, the exception being advanced treatment (0.608 versus 0.614).

**Table 4:** Subgroup analysis of mapping performance at k = 5, together with R-precision, for the human-only, Agent-only and Agent-assisted conditions, by number of SNOMED CT concepts per record and by clinical category

Subgroup	Condition	Hit Rate@5	Precision@5	Recall@5	F1@5	*R*-precision
**SNOMED CT concepts per record**						
Single concept	Human-only	0.840	0.168	0.840	0.280	0.830
	Agent-only	0.879	0.176	0.879	0.293	0.753
	Agent-assisted	0.885	0.177	0.885	0.295	0.870
Multiple concepts	Human-only	0.896	0.232	0.453	0.300	0.442
	Agent-only	0.868	0.307	0.596	0.395	0.492
	Agent-assisted	0.914	0.254	0.495	0.327	0.486
**Clinical category**						
Test	Human-only	0.868	0.200	0.655	0.290	0.645
	Agent-only	0.884	0.233	0.734	0.334	0.631
	Agent-assisted	0.877	0.207	0.675	0.301	0.667
Advanced procedure	Human-only	0.785	0.183	0.572	0.264	0.560
	Agent-only	0.832	0.237	0.692	0.333	0.556
	Agent-assisted	0.844	0.201	0.621	0.287	0.606
Advanced treatment	Human-only	0.881	0.227	0.632	0.312	0.614
	Agent-only	0.839	0.235	0.655	0.324	0.535
	Agent-assisted	0.878	0.205	0.621	0.290	0.608
Underlying disease	Human-only	0.858	0.179	0.712	0.278	0.707
	Agent-only	0.906	0.233	0.835	0.349	0.711
	Agent-assisted	0.919	0.204	0.782	0.312	0.777
Lesion characteristics	Human-only	0.856	0.208	0.600	0.289	0.576
	Agent-only	0.901	0.263	0.717	0.359	0.611
	Agent-assisted	0.886	0.225	0.638	0.311	0.619
Medication	Human-only	0.932	0.203	0.632	0.295	0.632
	Agent-only	0.824	0.252	0.724	0.361	0.636
	Agent-assisted	0.931	0.238	0.708	0.343	0.699
Recurrent disease	Human-only	0.924	0.220	0.664	0.312	0.660
	Agent-only	0.900	0.269	0.746	0.371	0.625
	Agent-assisted	0.927	0.235	0.685	0.329	0.667
Symptom	Human-only	0.921	0.202	0.734	0.305	0.730
	Agent-only	0.908	0.233	0.812	0.347	0.688
	Agent-assisted	0.921	0.214	0.765	0.321	0.752
Medical history	Human-only	0.794	0.187	0.582	0.266	0.561
	Agent-only	0.869	0.232	0.694	0.327	0.585
	Agent-assisted	0.915	0.216	0.680	0.309	0.672

Across clinical categories, symptom-related texts achieved the highest Agent-only hit rate (0.908), recurrent disease the highest Agent-only precision (0.269) and F1 (0.371), and underlying disease the highest Agent-only recall (0.835); medication showed the lowest Agent-only hit rate (0.824). Under the Agent-assisted condition the highest hit rate was observed for medication (0.931) and the lowest for advanced procedure (0.844). The largest Agent-assisted gains in R-precision over human-only mapping were in medical history (+ 0.111), underlying disease (+ 0.070), and medication (+ 0.067).

## Discussion

In this study, the agent-assisted human strategy achieved a pooled hit rate@1 of 0.868 across 2,261 clinical text segments, outperforming both the Agent-only approach (0.701) and unassisted human mapping (0.837), and reduced mean mapping time by 53.9%, from 1.57 to 0.72 min per segment, including the agent’s own processing. Beyond the accuracy gains reported for LLM-based terminology mapping [24], these results show that the practical value of such systems extends to efficiency in real-world workflows. Because reliable concept selection requires expert navigation of SNOMED CT’s hierarchical and attribute relationships using the SNOMED CT Browser (Supplementary Material 2) [2], the system was designed to augment rather than replace this workflow. Notably, 21% of selected concepts were identified independently through the Browser, showing that mappers made decisions beyond the agent’s suggestions.

A principal finding was that expanding the space of valid SNOMED CT candidates substantially improved mapping accuracy: mappers identified correct concepts they might not otherwise retrieve in the conventional Browser workflow, raising pooled hit rate@1 from 0.837 to 0.868. The expanded candidate space allowed mappers to select different but individually valid concepts when more than one correct answer existed; agreement between mappers declined under assistance in both strata (single-concept 0.969 to 0.836; multi-concept 0.862 to 0.450), and previous studies have likewise reported considerable disagreement among SNOMED CT coding experts [9]. A recent study on human-AI collaborative coding for the International Classification of Diseases (ICD) similarly reported that AI-generated pre-codes reduced cognitive burden and supported expert decision-making [25]. Our study provides one of the first systematic evaluations of such a workflow in a dedicated SNOMED CT context, outperforming both fully automated and manual-only strategies [26].

Recent advances in LLMs have accelerated automated approaches to clinical terminology mapping: a scoping review identified 37 studies of LLM-based terminology mapping tasks [24], retrieval-augmented generation (RAG)-enhanced approaches have improved drug-term mapping and SNOMED CT entity linking over standalone LLMs [27, 28], and the SNOMED CT Entity Linking Challenge established benchmarks, with performance varying by concept frequency and text complexity [13]. Our approach differs in scope: whereas most prior studies focused on specific concept types, such as drug terms [27] or limited subsets of body structure, procedure and clinical finding [13], our evaluation encompassed nine clinical categories ranging from symptoms and diagnoses to medications and surgical procedures. In the subgroup analysis, the LLM agent showed similar performance across different categories, supporting the generalizability of our system.

Our system employs a pre-embedded SNOMED CT vector database that operates entirely within a local environment, requiring no external application programming interface (API) calls during retrieval. Unlike BERT-based entity linking that requires training on labeled clinical notes [13], this design needs no supervised learning: because the SNOMED CT International Edition is updated monthly, mapping capability is maintained by periodically refreshing the vector database rather than retraining, and the RAG approach itself has been shown to reduce hallucinations and improve grounding in medical LLMs [29]. The retrieval component therefore suits hospitals where patient data must remain on-premises; the translation and abbreviation modules currently call an external LLM API, and a locally hosted model would be required for a fully on-premises deployment. The prompt-based architecture can be adapted to specific specialties or documentation styles by modifying prompts, and the Translation Module provides bilingual capability, addressing a recognized gap in tools designed primarily for English-language text [30].

The 53.9% reduction in overall mapping time (from 59.2 to 27.3 h for 2,261 segments) has practical implications for terminology standardization programs, where manual coding is a major resource bottleneck. This aligns with evidence that AI-assisted tools reduce clinician documentation time [31], and AI-assisted ICD coding in hospital settings has likewise shown time savings while maintaining accuracy [32]. Notably, the time reduction was consistent across all nine clinical categories (35.8%–64.6%), suggesting that the efficiency benefit generalizes across diverse clinical content types.

This study has several limitations. First, the evaluation was conducted at a single institution using discharge and surgical records, which may not fully represent the diversity of clinical documentation across healthcare settings, and although the system performed well in the Korean-English bilingual context, generalizability to other language pairs warrants separate investigation. Second, the evaluator pool was limited to three mappers, and broader evaluation with additional independent coders across institutions would test whether these findings hold beyond this setting. Each segment was mapped in isolation, without the surrounding clinical record that a coder would ordinarily consult; this keeps the comparison between conditions clean, but the context-free design may underestimate real-world performance variability, because the wider record can both resolve and introduce ambiguity. The observations are also clustered: the 2,261 segments derive from 2,009 discharge cases and 1,763 distinct patients, so 20.7% of segments share a case with at least one other segment, and 246 of the 2,009 cases (12.2%) come from a patient who contributed more than one case. Third, the translation and abbreviation modules rely on proprietary LLM APIs; open-source alternatives would be necessary under strict data governance. Fourth, mapping time was timestamped automatically in the Agent-assisted condition but self-reported in the human-only condition; this asymmetry introduces measurement error, and follow-up evaluations should use automated time capture in all arms. Fifth, the reference panel and the mappers shared an institution and mapping guidance, so their errors may not be conditionally independent; where errors correlate, measured accuracy is biased upward and should be read as a plausible upper estimate. Sixth, both conditions were performed in a fixed order without counterbalancing, so the Agent-assisted session was not independent of the earlier one; the sessions were separated by a wash-out interval of approximately four months, but residual influence cannot be excluded, and a counterbalanced design would isolate the agent’s contribution more cleanly. Seventh, the fixed-cutoff metrics are bounded by the number of reference concepts a segment carries (macro-averaged recall@1 cannot exceed 0.698 on this dataset even for a perfect mapping) and therefore quantify differences between conditions; absolute performance is measured by R-precision, whose per-segment cutoff keeps the attainable maximum at 1.0. Eighth, because capitalization is presented as a positive cue, the Abbreviation Expansion Module is biased toward capitalized forms, with lowercase abbreviations identical to common English words (for example, “bid” or “who”) the hardest case; per-module performance was not evaluated separately and would be analyzed in future studies.

Future research should include multi-center and prospective validation beyond this single tertiary hospital, extension to additional language pairs, and replacement of proprietary LLM APIs with open-source alternatives under strict data governance requirements. Performance with complex or nested abbreviations and the value of user feedback mechanisms warrant study.

## Conclusion

We developed and evaluated a human-AI collaborative approach for SNOMED CT coding of bilingual clinical text. By expanding the space of valid SNOMED CT candidates available to expert mappers, the agent-assisted workflow improved mapping accuracy while reducing coding time by about half compared with unassisted manual mapping. The system’s modular architecture, supporting periodic vector database updates without retraining, offers a sustainable and adaptable solution for clinical terminology standardization.

The Abbreviation Expansion Module identifies and expands domain-specific abbreviations and acronyms by leveraging category and department metadata. For example, “PCI” is expanded to “Percutaneous Coronary Intervention” when the department is Cardiology, but may be expanded differently in other clinical contexts. The module identifies potential abbreviations by treating capitalization as a probabilistic cue rather than a filter, so lowercase and mixed-case abbreviations are also expanded, and uses LLM-based prompts augmented with web search tools to retrieve context-appropriate full forms when the category and department metadata alone do not determine the expansion. The complete system prompt, which directs the module to expand abbreviations into the form “Expanded Phrase (ABBR)” rather than narrative interpretations, is provided in Supplementary Material 1.

## Supplementary Information

Below is the link to the electronic supplementary material.

Supplementary File 1 (DOCX 29.0 KB)

Supplementary File 2 (DOCX 523 KB)

Supplementary File 3 (DOCX 23.6 KB)

Supplementary File 4 (DOCX 25.2 KB)

Supplementary File 5 (DOCX 23.9 KB)

## Data Availability

No datasets were generated or analysed during the current study.

## Abbreviations

AI: artificial intelligence
API: application programming interface
BERT: bidirectional encoder representations from transformers
CI: confidence interval
F1: F1-score
GPT-4: generative pre-trained transformer 4
ICD: International Classification of Diseases
IQR: interquartile range
IRB: institutional review board
KHIDI: Korea Health Industry Development Institute
LLM: large language model
OMOP: Observational Medical Outcomes Partnership
PCI: percutaneous coronary intervention
RAG: retrieval-augmented generation
SIMS: SNUH Integrated Indicator Management System
SNOMED CT: Systematized Nomenclature of Medicine–Clinical Terminology
SNUH: Seoul National University Hospital

## References

[1] Chang E, Mostafa J. The use of SNOMED CT, 2013–2020: a literature review. J Am Med Inform Assoc. 2021;28(9):2017–2026. 10.1093/jamia/ocab084. PMID: 34151978. PMC8363812 34151978

[2] Cornet R, de Keizer N. Forty years of SNOMED: a literature review. BMC Med Inform Decis Mak. 2008;8(Suppl 1):S2. 10.1186/1472-6947-8-S1-S2. PMID: 19007439. PMC2582789 19007439

[3] Sung S, Park HA, Jung H, Kang H. A SNOMED CT mapping guideline for the local terms used to document clinical findings and procedures in electronic medical records in South Korea: methodological study. JMIR Med Inform. 2023;11:e46127. 10.2196/46127. PMID: 37071456. PMC10155087 37071456

[4] Park HA. Why terminology standards matter for data-driven artificial intelligence in healthcare. Ann Lab Med. 2024;44(6):467–471. 10.3343/alm.2024.0105. PMID: 38955364. PMC11375201 38955364

[5] Wang L, Ma Y, Bi W, Lv H, Li Y. An entity extraction pipeline for medical text records using large language models: analytical study. J Med Internet Res. 2024;26:e54580. 10.2196/54580. PMID: 38551633. PMC11015372 38551633

[6] Richesson RL, Andrews JE, Krischer JP. Use of SNOMED CT to represent clinical research data: a semantic characterization of data items on case report forms in vasculitis research. J Am Med Inform Assoc. 2006;13(5):536–546. 10.1197/jamia.M2093. PMID: 16799121. PMC1561787 16799121

[7] Balch JA, Ruppert MM, Loftus TJ, et al. Machine learning-enabled clinical information systems using fast healthcare interoperability resources data standards: scoping review. JMIR Med Inform. 2023;11:e48297. 10.2196/48297. PMID: 37646309. PMC10468818 37646309

[8] Minarro-Gimenez JA, Martinez-Costa C, Karlsson D, Schulz S, Goeg KR. Qualitative analysis of manual annotations of clinical text with SNOMED CT. PLoS One. 2018;13(12):e0209547. 10.1371/journal.pone.0209547. PMID: 30589855. PMC6307753 30589855

[9] Andrews JE, Richesson RL, Krischer J. Variation of SNOMED CT coding of clinical research concepts among coding experts. J Am Med Inform Assoc. 2007;14(4):497–506. 10.1197/jamia.M2372. PMID: 17460128. PMC2244907 17460128

[10] Schwab JD, Werle SD, Huhne R, Spohn H, Kaisers UX, Kestler HA. The necessity of interoperability to uncover the full potential of digital health devices. JMIR Med Inform. 2023;11:e49301. 10.2196/49301. PMID: 38133917. PMC10770786 38133917

[11] Ochs C, Geller J, Perl Y, et al. Scalable quality assurance for large SNOMED CT hierarchies using subject-based subtaxonomies. J Am Med Inform Assoc. 2015;22(3):507–518. 10.1136/amiajnl-2014-003151. PMID: 25336594. PMC6283060 25336594

[12] Kang B, Yoon J, Kim HY, Jo SJ, Lee Y, Kam HJ. Deep-learning-based automated terminology mapping in OMOP-CDM. J Am Med Inform Assoc. 2021;28(7):1489–1496. 10.1093/jamia/ocab030. PMID: 33987667. PMC8279781 33987667

[13] Davidson R, Hardman W, Amit G, et al. SNOMED CT entity linking challenge. J Am Med Inform Assoc. 2025;32(9):1397–1406. 10.1093/jamia/ocaf104. PMID: 40657868. PMC12361850 40657868

[14] Fan H, Rossetti SC, Thate J, Mugoya R, Lai AM, Yen PY. Semi-automated pipeline to accelerate multi-site flowsheet alignment and concept mapping in electronic health records. J Am Med Inform Assoc. 2025;32(7):1140–1148. 10.1093/jamia/ocaf076. PMID: 40378254. PMC12202140 40378254

[15] Puts S, Zegers CML, Dekker A, Bermejo I. Developing an ICD-10 coding assistant: pilot study using RoBERTa and GPT-4 for term extraction and description-based code selection. JMIR Form Res. 2025;9:e60095. 10.2196/60095. PMID: 39935026. PMC11835781 39935026

[16] Abeysinghe R, Zheng F, Bernstam EV, Shi J, Bodenreider O, Cui L. A deep learning approach to identify missing is-a relations in SNOMED CT. J Am Med Inform Assoc. 2023;30(3):475–484. 10.1093/jamia/ocac248. PMID: 36539234. PMC9933066 36539234

[17] Kim S, Choi B, Lee K, Lee S, Kim S. Assessing the performance of a method for case-mix adjustment in the Korean Diagnosis-Related Groups (KDRG) system and its policy implications. Health Res Policy Syst. 2021;19(1):98. 10.1186/s12961-021-00739-5. PMID: 34187515. PMC8243480 34187515

[18] SNOMED International. SNOMED CT International Edition, release 2024-08-01. London: SNOMED International; 2024.

[19] Chroma. Chroma: the open-source AI application database. https://www.trychroma.com (accessed 13 August 2026).

[20] Jin Q, Kim W, Chen Q, et al. MedCPT: contrastive pre-trained transformers with large-scale PubMed search logs for zero-shot biomedical information retrieval. Bioinformatics. 2023;39(11):btad651. 10.1093/bioinformatics/btad651. PMID: 37930897. PMC10627406 37930897

[21] Manning CD, Raghavan P, Schütze H. Introduction to Information Retrieval. Cambridge: Cambridge University Press; 2008.

[22] Buckley C, Voorhees EM. Evaluating evaluation measure stability. In: Proceedings of the 23rd Annual International ACM SIGIR Conference on Research and Development in Information Retrieval. New York: ACM; 2000. p. 33–40.

[23] Yuan X, Wang T, Meng R, et al. One size does not fit all: generating and evaluating variable number of keyphrases. In: Proceedings of the 58th Annual Meeting of the Association for Computational Linguistics; 2020. 10.18653/v1/2020.acl-main.710.

[24] Chang E, Sung S. Use of SNOMED CT in large language models: scoping review. JMIR Med Inform. 2024;12:e62924. 10.2196/62924. PMID: 39374057. PMC11494256 39374057

[25] Gao Y, Chen Y, Wang M, et al. Optimising the paradigms of human AI collaborative clinical coding. NPJ Digit Med. 2024;7(1):368. 10.1038/s41746-024-01363-7. PMID: 39702575. PMC11659570 39702575

[26] Adams MCB, Perkins ML, Hudson C, et al. Breaking digital health barriers through a large language model-based tool for automated Observational Medical Outcomes Partnership mapping: development and validation study. J Med Internet Res. 2025;27:e69004. 10.2196/69004. PMID: 40146872. PMC12123247 40146872

[27] Kimura E, Kawakami Y, Inoue S, Okajima A. Mapping drug terms via integration of a retrieval-augmented generation algorithm with a large language model. Healthc Inform Res. 2024;30(4):355–363. 10.4258/hir.2024.30.4.355. PMID: 39551922. PMC11570653 39551922

[28] Berkowitz JS, Srinivasan A, Acitores Cortina JM, Fatapour Y, Tatonetti NP. Biomedical text normalization through generative modeling. J Biomed Inform. 2025;167:104850. 10.1016/j.jbi.2025.104850. PMID: 40381869. PMC12158622 40381869

[29] Liu S, McCoy AB, Wright A. Improving large language model applications in biomedicine with retrieval-augmented generation: a systematic review, meta-analysis, and clinical development guidelines. J Am Med Inform Assoc. 2025;32(4):605–615. doi:10.1093/jamia/ocaf008. PMID: 39812777. PMC12005634 39812777

[30] Bedi S, Liu Y, Orr-Ewing L, et al. Testing and evaluation of health care applications of large language models: a systematic review. JAMA. 2025;333(4):319–328. 10.1001/jama.2024.21700. PMID: 39405325. PMC11480901 39405325

[31] Duggan MJ, Gervase J, Schoenbaum A, et al. Clinician experiences with ambient scribe technology to assist with documentation burden and efficiency. JAMA Netw Open. 2025;8(2):e2460637. 10.1001/jamanetworkopen.2024.60637. PMID: 39969880. PMC11840636 39969880

[32] Dai HJ, Wang CK, Chen CC, et al. Evaluating a natural language processing-driven, AI-assisted International Classification of Diseases, 10th Revision, Clinical Modification coding system for diagnosis related groups in a real hospital environment: algorithm development and validation study. J Med Internet Res. 2024;26:e58278. 10.2196/58278. PMID: 39302714. PMC11452756 39302714

