# Supplementary material for: Development and Validation of Human-AI Collaborative Workflow in SNOMED CT Mapping of Bilingual Clinical Text
Source: J Med Syst. 2026 Oct 2;50(1):141. doi: 10.1007/s10916-026-02465-3 (PMC13633333; doi:10.1007/s10916-026-02465-3)
Supplement: Supplementary file 2 [file 10916_2026_2465_MOESM2_ESM.docx]

# **Supplementary Material 2. SNOMED CT structure and finding concepts using browser**

SNOMED CT is a core clinical healthcare terminology that contains concepts with unique meanings and formally defined logical definitions, organized into hierarchies. SNOMED CT content is represented using three types of components: Concepts, Descriptions, and Relationships.
Concepts represent clinical meanings that are structured into hierarchies (domains). A concept can have several associated descriptions, each representing a synonym that describes the same clinical concept. SNOMED CT relationships link concepts to other concepts whose meanings are related in some way. Relationships are used to logically define the meaning of a concept in a manner that can be processed by a computer. One type of relationship is the "Is-a" relationship, which relates a concept to more general concepts. These "Is-a" relationships define the hierarchy of SNOMED CT concepts. Another type of relationship is the "Attribute Relationship." An attribute relationship contributes to the definition of the source concept by associating it with the value of a defining characteristic. The characteristic (attribute) is specified by the relationship type, and the value is provided by the destination of the relationship. A Concept consists of a unique concept identifier (ID) and a Fully Specified Name (FSN). The FSN is derived from a description. The Fully Specified Name (FSN) and synonyms are description types in SNOMED CT. An FSN is unique among active descriptions in SNOMED CT. It provides the meaning of a concept so that it remains unambiguous, stable across multiple contexts, and optimally understandable. A Semantic Tag is part of an FSN. It is placed in parentheses at the end of an FSN when authoring concepts. Semantic tags indicate the domain to which a concept belongs, for example body structure, disorder, or specimen. The purpose of semantic tags is to disambiguate concepts that share the same commonly used word or phrase.

For example,
122488002 |Transection of vas deferens(procedure)|
262920002 |Transection of vas deferens(disorder) |

The concept '122488002 |Transection of vas deferens (procedure)|' refers to a procedure involving surgical cutting (transversely across a long axis) of the vas deferens. In contrast, '262920002 |Transection of vas deferens (disorder)|' describes a condition in which the vas deferens has been transected. Although both concepts share the same terminology, their semantic tags assign them entirely different meanings.

The following describes the fundamental process in which a mapper searches for an appropriate concept using the SNOMED CT browser and verifies its meaning through the core structure of SNOMED CT.

| **Steps** | **Reference** |
| --- | --- |
| As indicated above the search box, the system allows searches using at least the first three letters of each word. For example, when searching for the disease Heart Failure, entering "hear" from the first word and "fail" from the second word will retrieve descriptions (FSN and synonyms) that include the corresponding text. |  |
| The filtering function based on semantic tags allows for the extraction of only the necessary semantic tags from the search results. Semantic tags can be filtered according to the specific clinical situation or medical context to be mapped. |  |
| The 'Summary' section provides detailed information about the retrieved concept. It includes the fundamental structure of SNOMED CT, which consists of concepts and their various synonymous expressions (Descriptions).  The upper and lower sections of a concept represent its parent and child concepts within the 'Is-a Relationship' hierarchy, while the right side displays the 'Attribute Relationship' that specifies the retrieved concept.  By analyzing these Descriptions and Relationships, users can semantically verify whether the retrieved concept aligns with the intended target. |  |
