# Supplementary material for: Development and Validation of Human-AI Collaborative Workflow in SNOMED CT Mapping of Bilingual Clinical Text
Source: J Med Syst. 2026 Oct 2;50(1):141. doi: 10.1007/s10916-026-02465-3 (PMC13633333; doi:10.1007/s10916-026-02465-3)
Supplement: Supplementary file 3 [file 10916_2026_2465_MOESM3_ESM.docx]

**Supplementary Material 5. Agent processing time, by stage**

| **Stage** | **Mean (s)** | **95% CI** | **Median (s)** | **IQR (s)** |
| --- | --- | --- | --- | --- |
| Translation Module (LLM) | 6.50 | 5.41–7.66 | 5.75 | 1.89–9.31 |
| Abbreviation Expansion Module (LLM) | 3.33 | 2.80–3.96 | 2.42 | 2.00–2.93 |
| MedCPT query encoding | 0.04 | 0.03–0.05 | 0.03 | 0.03–0.04 |
| ChromaDB retrieval (top-20) | 1.56 | 1.52–1.61 | 1.64 | 1.33–1.72 |
| **End-to-end per segment** | **11.44** | **10.23–12.74** | **10.63** | **6.26–13.81** |
