# Supplementary material for: Development and Validation of Human-AI Collaborative Workflow in SNOMED CT Mapping of Bilingual Clinical Text
Source: J Med Syst. 2026 Oct 2;50(1):141. doi: 10.1007/s10916-026-02465-3 (PMC13633333; doi:10.1007/s10916-026-02465-3)
Supplement: Supplementary file 4 [file 10916_2026_2465_MOESM4_ESM.docx]

**Supplementary Material 4. Pairwise comparisons of mapping approaches using paired t-tests.**

| **Comparison** | **Metric** | **k** | **Mean Difference** | **p-value** |
| --- | --- | --- | --- | --- |
| Agent-only vs. Human-only | Hit Rate | 1 | -0.135 (-0.148 to -0.123) | <0.001 |
| Agent-only vs. Human-only | Precision | 1 | -0.135 (-0.148 to -0.123) | <0.001 |
| Agent-only vs. Human-only | Recall | 1 | -0.076 (-0.085 to -0.067) | <0.001 |
| Agent-only vs. Human-only | F1 | 1 | -0.092 (-0.102 to -0.083) | <0.001 |
| Agent-assisted vs. Human-only | Hit Rate | 1 | 0.031 (0.021 to 0.042) | <0.001 |
| Agent-assisted vs. Human-only | Precision | 1 | 0.031 (0.021 to 0.042) | <0.001 |
| Agent-assisted vs. Human-only | Recall | 1 | 0.024 (0.016 to 0.031) | <0.001 |
| Agent-assisted vs. Human-only | F1 | 1 | 0.025 (0.017 to 0.034) | <0.001 |
| Agent-assisted vs. Agent-only | Hit Rate | 1 | 0.167 (0.156 to 0.177) | <0.001 |
| Agent-assisted vs. Agent-only | Precision | 1 | 0.167 (0.156 to 0.177) | <0.001 |
| Agent-assisted vs. Agent-only | Recall | 1 | 0.100 (0.093 to 0.108) | <0.001 |
| Agent-assisted vs. Agent-only | F1 | 1 | 0.118 (0.110 to 0.126) | <0.001 |
| Agent-only vs. Human-only | Hit Rate | 5 | 0.005 (-0.006 to 0.015) | 1.000 |
| Agent-only vs. Human-only | Precision | 5 | 0.042 (0.038 to 0.046) | <0.001 |
| Agent-only vs. Human-only | Recall | 5 | 0.092 (0.082 to 0.102) | <0.001 |
| Agent-only vs. Human-only | F1 | 5 | 0.055 (0.050 to 0.060) | <0.001 |
| Agent-assisted vs. Human-only | Hit Rate | 5 | 0.031 (0.021 to 0.040) | <0.001 |
| Agent-assisted vs. Human-only | Precision | 5 | 0.015 (0.012 to 0.019) | <0.001 |
| Agent-assisted vs. Human-only | Recall | 5 | 0.044 (0.035 to 0.052) | <0.001 |
| Agent-assisted vs. Human-only | F1 | 5 | 0.021 (0.017 to 0.026) | <0.001 |
| Agent-assisted vs. Agent-only | Hit Rate | 5 | 0.026 (0.017 to 0.035) | <0.001 |
| Agent-assisted vs. Agent-only | Precision | 5 | -0.027 (-0.030 to -0.023) | <0.001 |
| Agent-assisted vs. Agent-only | Recall | 5 | -0.048 (-0.057 to -0.039) | <0.001 |
| Agent-assisted vs. Agent-only | F1 | 5 | -0.034 (-0.039 to -0.029) | <0.001 |
| Agent-only vs. Human-only | R-precision | g | -0.012 (-0.022 to -0.002) | 0.070 |
| Agent-assisted vs. Human-only | R-precision | g | 0.042 (0.034 to 0.051) | <0.001 |
| Agent-assisted vs. Agent-only | R-precision | g | 0.054 (0.045 to 0.063) | <0.001 |

For R-precision, the cutoff g equals each segment's number of reference concepts; the p-value for the Agent-only versus human-only comparison is Bonferroni-corrected, as elsewhere in the table.
