# Supplementary material for: Development and Validation of Human-AI Collaborative Workflow in SNOMED CT Mapping of Bilingual Clinical Text
Source: J Med Syst. 2026 Oct 2;50(1):141. doi: 10.1007/s10916-026-02465-3 (PMC13633333; doi:10.1007/s10916-026-02465-3)
Supplement: Supplementary file 5 [file 10916_2026_2465_MOESM5_ESM.docx]

**Supplementary Material 3. Length of the clinical text segments, by clinical category**

| **Clinical category** | **n** | **Characters, median (IQR)** | **Characters, range** | **Words, median (IQR)** |
| --- | --- | --- | --- | --- |
| Test | 250 | 10 (7–15) | 2–40 | 2 (1–3) |
| Advanced procedure | 250 | 16 (9–21) | 2–59 | 2 (2–3) |
| Advanced treatment | 254 | 13 (9–19) | 3–56 | 2 (2–3) |
| Underlying disease | 254 | 12 (6–18) | 2–55 | 2 (1–3) |
| Lesion characteristics | 252 | 14 (8–20) | 2–67 | 2 (2–3) |
| Medication | 250 | 10 (7–14) | 2–33 | 1 (1–2) |
| Recurrent disease | 250 | 11 (6–16) | 2–55 | 2 (1–2) |
| Symptom | 250 | 13 (8–18) | 2–39 | 2 (1–3) |
| Medical history | 251 | 10 (7–17) | 2–55 | 2 (1–3) |
| **Overall** | **2,261** | **12 (7–18)** | **2–67** | **2 (1–3)** |
